# Effects of a Complex Intervention Exercise Program on Lumbar Extension Strength and Stability in Female Patients with Lower Back Pain

**Published:** 2017-06

**Authors:** Soonyoung KIM, Kyoungkyu JEON

**Affiliations:** 1. Dept. of Physical Education, Gachon University, Gyeonggi-Do, Republic of Korea; 2. Sport Science Institute, Incheon National University, Incheon, Republic of Korea; 3. Division of Sport Science, Incheon National University, Incheon, Republic of Korea

## Dear Editor-in-Chief

The lack of physical activity among adults in modern society can cause rigidness in posture, which in turn causes degeneration and chronic lower back pain owing to damage in the lumbar area, instability, and functional restrictions (1, 2). Exercise-based treatments are especially adopted to improve spinal cord mobility, muscle strength, flexibility, and stability (3). In particular, vertebral stability is an important factor for the prevention of acute and chronic lower back pain, and it has been shown to regulate bodily functions efficiently by positively affecting movement regulation, dynamic balance, and the neutral regulation ability of the vertebra (4, 5). Therefore, there is a need to identify the actual effects of an 8 wk program of exercise-based rehabilitation treatments that aim to improve functionally chronic lower back pain by enhancing complex body functions based on muscle strength and stability in order to develop effective exercise programs for patients with lower back pain.

This study was conducted in sport biomechanics laboratory of Incheon National University, Incheon, South Korea in 2016. We included 23 female patients (28.3±5.3 yr, 162.2±5.3 cm, 53.8±5.3 kg) who voluntarily participated after being diagnosed with acute or chronic lower back pain resulting from long duration of sitting during work.

All the subjects understood the purpose of this study and provided their written informed consent prior to a participation in the study in accordance with the ethical standards of the Declaration of Helsinki.

Lumbar muscle strength was evaluated using the Lumbar Extension Strength System (MedX Inc., Ocala, FL), which works under isometric principles. The range of lumbar movement (0–72 °) was checked before measurement, and the maximum lumbar extension strength was measured at the following angles. Physical stability was evaluated using the Tetrax multiple systems (Tetrax Ltd., Ramat Gan, Israel), and measurements were taken for about 32 sec, with the subject standing upright over a force plate. The foot was divided into 4 regions and the center of pressure (COP) was measured by using weight distribution at the forefoot and rear foot. The measured COP was set to 0, and the ideal weight at each region of the foot was set to 25%. The distribution of pressure across the foot was summed and was defined as the weight distribution index. The subjects underwent a 60 min complex intervention exercise program twice a week for 8 wk in order to relieve pain, enhance function, and improve muscle strength and physical stability. Cycling and stretching were performed for 10 min before and after the main exercise program as a warm up/cool down routine. The main exercise program included muscle strength and stability exercises using MedX (lumbar extension strength), weight machine (leg extension and raise, seated leg curl, internal and external torso, and extension and rotation of trunk), and other small equipment (balance ball, Swiss ball, foam roller).

The mean and standard deviation were calculated for every measurement category, and the paired *t-*test was used to analyze changes in every variable after the 8 wk complex intervention exercise program. All statistical analyses were performed using SPSS 23.0 (SPSS Inc., Chicago, IL). A *P*-value <0.05 was considered statistically significant.

The lumbar extension strengths significantly improved after the 8 wk complex exercise program than before the program at all assessed angles, and stability significantly improved only at the right rear foot (Table 1). This result suggests that resistance exercises based on stability should be actively performed by patients with lower back pain for effective rehabilitation.

**Table 1:** Changes in lumbar extension strength and stability

**Variables**		**Pre-test**	**Post-test**
Lumbar extension strength (ft-lbs)	0	47.5±16.7	73.1±18.5^**^
	12	62.3±18.1	92.0±20.1^***^
	24	71.7±20.2	107.7±19.5^***^
	36	83.5±20.0	121.7±22.5^***^
	48	91.9±20.5	129.0±21.7^***^
	60	98.6±21.8	140.0±23.5^***^
	72	105.6±24.2	151.0±27.9^***^
Stability (%)	Left fore-foot	23.1±4.6	23.3±6.6
	Left rear-foot	26.8±7.3	26.1±5.3
	Right fore-foot	23.0±4.7	23.4±6.7
	Right rear-foot	27.8±5.8	26.1±6.2^*^
	Weight distribution index	6.1±3.4	5.0±2.1

Values are Mean±SD,

* *P*<0.05,

** *P*<0.01,

*** *P*<0.001

Complex exercise programs might improve stability and relieve pain by positively affecting lumbar extension strength and stability in patients with lower back pain. Future studies should also consider coordination with lower limbs when developing exercise programs. Application of these exercise programs would improve functional performance in patients with lower back pain.
